# Design of a Reconfigurable Ultra-Wideband Terahertz Polarization Rotator Based on Graphene Metamaterial

**DOI:** 10.3390/s23125449

**Published:** 2023-06-08

**Authors:** Guowen Ding, Yanjun Zhou, Shuyang Zhang, Xinyao Luo, Shenyun Wang

**Affiliations:** 1 Research Center of Applied Electromagnetics, Nanjing University of Information Science and Technology, Nanjing 210044, China; gwding@nuist.edu.cn (G.D.);; 2 School of Electronic Science and Engineering, Nanjing University, Nanjing 210023, China

**Keywords:** graphene, metamaterial, polarization rotator

## Abstract

In this work, a reconfigurable ultra-wideband transmissive terahertz polarization rotator based on graphene metamaterial is proposed that can switch between two states of polarization rotation within a broad terahertz band by changing the Fermi level of graphene. The proposed reconfigurable polarization rotator is based on a two-dimensional periodic array of multilayer graphene metamaterial structure, which is composed of metal grating, graphene grating, silicon dioxide thin film, and a dielectric substrate. The graphene metamaterial can achieve high co-polarized transmission of a linearly polarized incident wave at the off-state of the graphene grating without applying the bias voltage. Once the specially designed bias voltage is applied to change the Fermi level of graphene, the polarization rotation angle of linearly polarized waves is switched to 45° by the graphene metamaterial at the on-state. The working frequency band with 45-degree linear polarized transmission remaining above 0.7 and the polarization conversion ratio (PCR) above 90% is from 0.35 to 1.75 THz, and the relative bandwidth reaches 133.3% of the central working frequency. Furthermore, even with oblique incidence at large angles, the proposed device retains high-efficiency conversion in a broad band. The proposed graphene metamaterial offers a novel approach for the design of a terahertz tunable polarization rotator and is expected to be applied in the applications of terahertz wireless communication, imaging, and sensing.

## 1. Introduction

Terahertz waves, which bridge the gap between macroscopic electronics and nanoscale photonics, are electromagnetic (EM) waves between millimeter waves and infrared light [1]. They have a wide range of applications in sensing [2], imaging [3], wireless communication [4,5,6], detection [7,8], and biological diagnosis [9].

Polarization control is an important aspect of free-space terahertz EM wave propagation. Polarization converters play an important role in terahertz polarization modulation [10]. Compared with the traditional methods based on birefringent crystals or the Faraday effect to achieve polarization conversion, the polarization converters based on metamaterials proposed in recent years have the advantages of small size and easy integration and are more suitable for achieving modulation in the terahertz band [11]. Metamaterials are artificial composites with special properties that are periodic or non-periodic. Metamaterials have some unique properties, such as negative refractive index, negative permittivity, negative magnetic permeability, etc. They are increasingly applied in a wide range of applications [12,13,14].

As one type of polarization converter, a polarization rotator is a device that can rotate the electric field vector of an incident linearly polarized EM wave by certain degrees. However, most reported metamaterial polarization rotators are passive devices that can’t change the polarization state of a terahertz wave on demand once the geometric parameters are fixed. Compared with passive metamaterial polarization rotators, the reconfigurable metamaterial polarization converter can provide more freedom and enables functional integration [11,15,16].

For designing reconfigurable devices in the terahertz band, loading active materials is a good candidate. Graphene is a two-dimensional active material with a thickness of one atomic layer, and it has excellent optical and electrical properties [17,18]. The electrical conductivity of single-layer graphene can be expressed by the Kubo formula [19]. Graphene can be obtained by chemical vapor deposition (CVD) [20]. Among various control techniques, voltage control is the simplest and most convenient method. The conductivity of graphene can be affected by the Fermi level, which can be controlled by an external bias voltage [21,22,23,24]. These properties make graphene an ideal material for designing reconfigurable polarization rotators.

At present, most reconfigurable polarization rotators mainly realize the conversion of orthogonal polarization waves, but other types of polarization rotation are less studied. In some communication systems, 45° polarized waves are necessary, and in some specific situations, this sort of polarization has more advantages than 0° or 90° polarization, such as symmetric propagation characteristics. As for some terahertz emitters, 45° linear polarization output is also needed in the system [25]. Therefore, it is necessary for further research on reconfigurable 45° polarization rotators that have application requirements in some scenarios.

In this work, a reconfigurable, high-efficiency, and ultra-wideband polarization rotator is proposed, which has a simple structure and consists of a metal wire grating, graphene gratings, an insulating film, and a dielectric substrate. By changing the bias voltage loaded on the graphene gratings, the polarization rotation angle of the transmitted linearly polarized wave can be easily switched between 0° and 45°. Moreover, the proposed polarization rotator is insensitive to oblique incidence. The proposed graphene-based metamaterial polarization rotator has excellent performance and will have potential applications in the fields of terahertz wireless communication, sensing, and imaging.

## 2. Element Design

**Figure 1:**
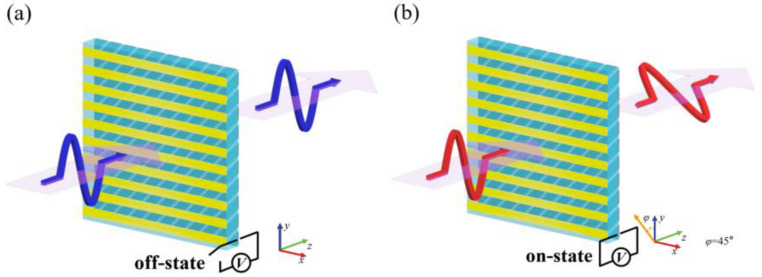
Schematic diagram of the polarization rotator: (**a**) graphene gratings are in the off-state; (**b**) graphene gratings are in the on-state.

Figure 1 shows the two working states of the reconfigurable polarization rotator. In order to achieve the tunable function of the polarization state, the polarization rotator’s slanted grating material was made of graphene. As shown in Figure 1a, when the graphene grating was in the off-state and the Fermi level of the graphene was located at the Dirac point, the incident terahertz wave was less affected by the graphene grating in the working frequency band. The on-state of the graphene grating is shown in Figure 1b, where the Fermi level of the graphene was shifted away from the Dirac point by applying a gate voltage, resulting in a change in the anisotropic graphene grating that caused the *y*-polarized waves to pass through the metal grating with a 45° polarization rotation [26].

**Figure 2:**
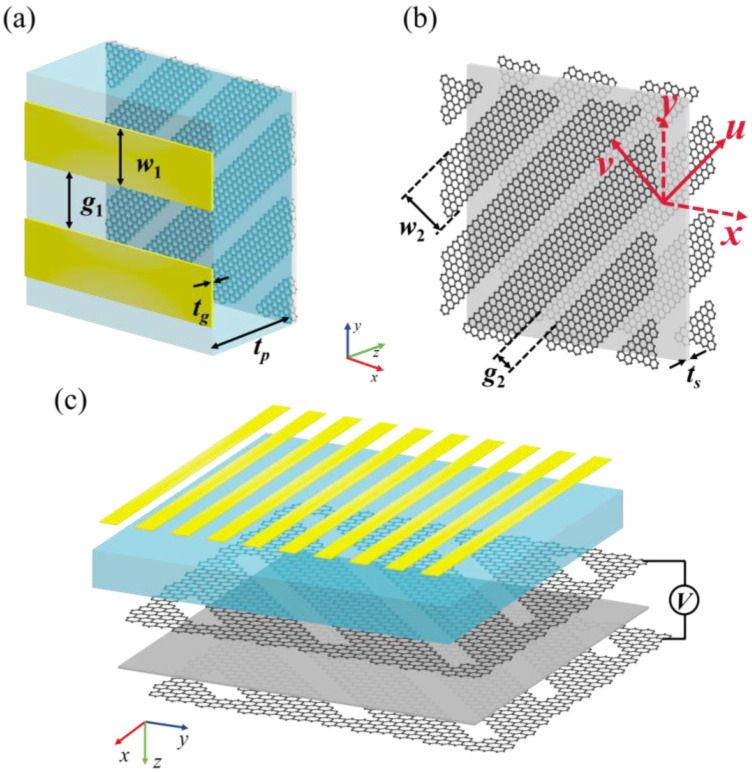
Schematic of unit structure of reconfigurable ultra-wideband terahertz polarization rotator based on graphene metamaterial: (**a**) three-dimensional view; (**b**) segregated schematic of graphene gratings and silicon dioxide insulating isolation layer; and (**c**) schematic of reconfigurable ultra-wideband terahertz polarization rotator and bias voltage loading.

The three-dimensional schematic diagram of the reconfigurable polarization rotator unit structure proposed in this paper is shown in Figure 2a. Along the *z*-axis of the three-dimensional coordinate system, the metal wire gratings, dielectric layer, first graphene grating layer, silicon dioxide insulating layer, and second graphene grating layer are arranged from front to back.

In order to pursue the maximization of the PCR, 45-degree linear polarized transmission, and the trade-off between the preservation of the PCR bandwidth in the considered frequency range and the material thinning, the element structure and structural parameters were optimized. The metal wire grating was parallel to the *x*-axis and composed of rectangular metal sheets arranged along the *y*-axis, with width *w*_1_ = 4 μm, thickness *t_m_* = 0.2 μm, and mutual interval *g*_1_ = 4 μm. The material was gold and has a conductivity *σ* = 4 × 10^7^ S/m, which is a perfect reflection layer in the terahertz band [19].

The thickness of the dielectric substrate layer was *t_p_* = 40 μm, and the material was TOPAS polymer with a relative dielectric constant of 2.35, which is an ideal terahertz dielectric substrate material due to its extremely low loss and low birefringence in the terahertz band [27]. As shown in Figure 2b, the first graphene grating layer and the second graphene grating layer were composed of two identical graphene gratings, with included angles of 45° relative to the *x*-axis. The graphene width *w*_2_ = 1.8 μm, and the mutual interval *g*_2_ = 3.8 μm. Between the two layers of graphene gratings was a silicon dioxide insulating isolation layer with height *t_s_* = 0.1 μm. Figure 2c shows the sandwich structure composed of graphene–silicon dioxide–graphene. The graphene grating layers served as the gate electrode, with one layer of graphene connected to the positive electrode and the other to the negative electrode [28].

The Kubo formula of the graphene layer can be expressed as follows:

(1) $$\sigma_{s}=\sigma_{\mathrm{intra}}(\omega ,E_{F},\Gamma ,T)+\sigma_{\mathrm{inter}}(\omega ,E_{F},\Gamma ,T)$$

(2) $$\sigma_{\mathrm{intra}}(\omega ,E_{F},\Gamma ,T)=-j\frac{e^{2}k_{B}T}{\pi \hbar (\omega -j2\Gamma )}(\frac{E_{F}}{k_{B}T}+2\ln(e^{-E_{F}/k_{B}T}+1))$$

(3) $$\sigma_{\mathrm{inter}}(\omega ,E_{F},\Gamma ,T)≃\frac{-je^{2}}{4\pi \hbar }\ln(\frac{2\left|E_{F}\right|-(\omega -j2\Gamma )\hbar }{2\left|E_{F}\right|+(\omega -j2\Gamma )\hbar })$$

where *ω* is the operating angular frequency; *E_F_* is the Fermi level; *Γ* is the scattering rate, *Γ*= *ℏ*/2*τ*; *τ* is the relaxation time; *T* is the room temperature (*T* = 300 K); *e* is the electron charge; parameter *ℏ* is the reduced Planck constant; and *k_B_* is the Boltzmann constant. The Fermi–Dirac distribution $f_{d}(\zeta )={\left[1+e^{\left(\zeta -E_{F})/(k_{B}T\right)}\right]}^{-1}$ and the carrier density can be derived from the formula:

(4) $$n_{s}=\frac{1}{\pi \hbar^{2}v_{F}{}^{2}}\int_{0}^{\infty }[f_{d}(\zeta )-f_{d}(\zeta +2E_{F})]\zeta d\zeta$$

where *ζ* is energy [29,30]. The mobility of graphene is *μ* = 2000 cm^2^/(Vs), which is derived from the formula: $\mu =\frac{\tau ev_{F}^{2}}{E_{F}}$, where the Fermi velocity $v_{F}\approx 1\times 10^{6}m/s$ [31]. The dispersion characteristics of graphene can be described by the thin layer’s dielectric constant, with thickness Δ [32]:.

(5) $$\varepsilon =\varepsilon_{0}+j\frac{\sigma_{s}}{\omega \Delta }$$

The graphene relaxation time *τ* of the reconfigurable polarization rotator in this paper was 0.2 ps, which is consistent with the measurement of graphene prepared by chemical vapor deposition (CVD). The Fermi level of graphene can be controlled by the bias voltage applied to it. The relationship between *E_F_* and *V_g_* can be expressed as [33]:

(6) $$E_{F}\approx \hbar v_{f}\sqrt{\frac{\pi \varepsilon_{r}\varepsilon_{0}V_{g}}{et_{s}}}$$

where *ε_r_* = 3.9 [34] is the relative permittivity of the insulating silicon dioxide film layer between two layers of graphene, and *ε*_0_ is the permittivity of vacuum. *V_g_* is the bias voltage; *e* is the electron charge; *v_f_* is the Fermi velocity, which is 1.1 × 10^6^ m/s; and *t_s_* is the thickness of the silicon dioxide insulating layer [35].

According to the relationship between the Fermi level *E_F_* and the bias voltage *V_g_*, *E_F_* = 0 when *V_g_* = 0, the conductivity of graphene is close to zero, and the graphene grating is in the off-state. As the bias voltage increased, the Fermi level of graphene increased. When the bias voltage was applied to meet the requirement of *E_F_* = 1 eV, the graphene grating was in the on-state. We performed a numerical simulation using commercial simulation software, HFSS. A planar periodic structure comprises identical unit cells; therefore, it is evaluated using Floquet ports and master–slave boundary conditions. The master–slave boundary conditions, also recognized as linked boundary conditions, are employed to simulate planar periodic structural surfaces. The graphene modeling approach relies on the zero-thickness modeling of the graphene layer, which is based on the application of an impedance boundary condition that accounts for the electrical response of the single-layer graphene, according to the Kubo formula.

**Figure 3:**
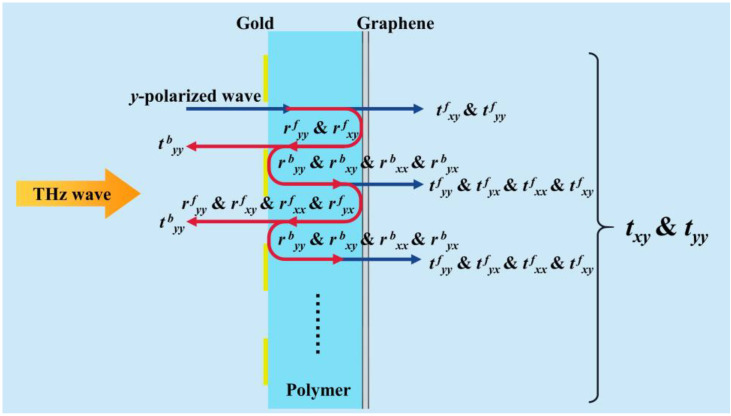
The schematic of the transmission process in the metamaterial.

In order to provide a better illustration, the polarization conversion characteristics of the Fabry–Perot-like cavity structure can be quantitatively analyzed using the multiple interference theory (MIT) [36]. As shown in Figure 3, the *y*-polarized incident EM waves illuminating the proposed structure were mostly transmitted into the polymer layer. Then, the transmitted *y*-polarized wave continued to propagate in the polymer layer with an additional phase factor and then reached the graphene grating layer. Due to the anisotropy of such a graphene grating layer, the EM wave was partially transmitted through the graphene grating, and the transmitted wave contains *x*- and *y*-polarized components. Meanwhile, the reflected wave also contained *x*- and *y*-polarized components and continued to propagate in the polymer layer until reaching the gold grating layer. Most of the EM wave was reflected again by this gold layer and propagated in the polymer layer. The above process was repeated, and the EM wave shuttled between the gold layer and graphene layer, thus forming multiple transmission and multiple reflection processes. The total transmission coefficient *t_xy_* to *t_yy_* of the proposed structure can be calculated by summing all cross-polarized and co-polarized transmitted waves.

For a single interface between two boundary media layers, A and B, the transfer matrix can be applied to describe the relationship between forward and backward propagating fields on either side of the interface, which is as follows:

(7) $$\left(\begin{array}{l} E_{Bx}^{}\\ E_{By}^{}\\ E_{Bx}^{}\\ E_{By}^{} \end{array}\right)=M_{BA}\left(\begin{array}{l} E_{Ax}^{}\\ E_{Ay}^{}\\ E_{Ax}^{}\\ E_{Ay}^{} \end{array}\right)$$

The transfer matrix *M_BA_* can be expressed as:

(8) $$M_{ba}={\left(\begin{array}{cccc} 1 & 0 & -r_{Bx;Bx} & -r_{Bx;By}\\ 0 & 1 & -r_{By;Bx} & -r_{By;By}\\ 0 & 0 & t_{Ax;Bx} & t_{Ax;By}\\ 0 & 0 & t_{Ay;Bx} & t_{Ay;By} \end{array}\right)}^{-1}\left(\begin{array}{cccc} t_{Bx;Ax} & t_{Bx;Ay} & 0 & 0\\ t_{By;Ax} & t_{By;Ay} & 0 & 0\\ -r_{Ax;Ax} & -r_{Ax;Ay} & 1 & 0\\ -r_{Ay;Ax} & -r_{Ay;Ay} & 0 & 1 \end{array}\right)$$

The subscripts *x* and *y* denote the polarization states of the EM waves in the medium layers A and B, while the superscripts *f* and *b* signify forward and backward propagation. Moreover, *r* and *t* represent the reflection and transmission coefficients, respectively. For a structure composed of several interfaces surrounded by homogeneous media, the overall transfer matrix is obtained by cascading multiple transfer matrices. Finally, the total transmission of the *x*-polarized and *y*-polarized waves with multiple interferences can be expressed as:

(9) $$E_{t,x\;\mathrm{or}\;y}=\sum_{j=1}^{\infty }E_{t,x\;\mathrm{or}\;y\;j}$$

where *j* is the number of the roundtrips within the spacer layer.

## 3. Results and Discussion

### 3.1. Polarization Rotation of Terahertz Waves

**Figure 4:**
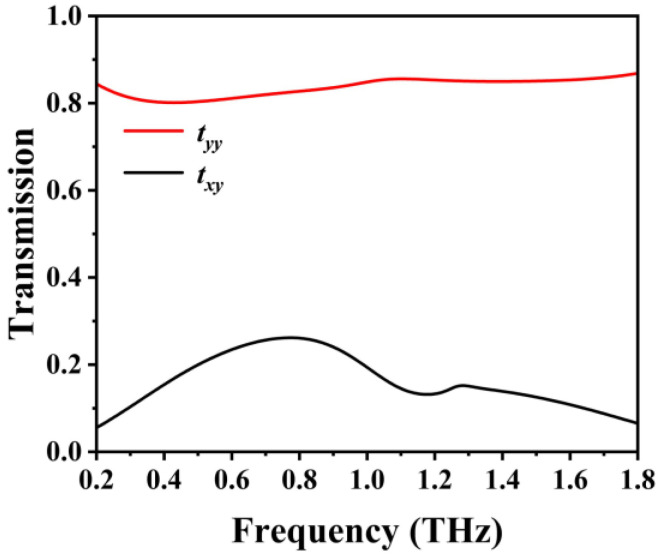
Transmittance of the co-polarization(*t_yy_*) and the cross-polarization(*t_xy_*) with *y*-polarized incident waves in the off-state.

Figure 4 depicts the HFSS-simulated transmission characteristic of the proposed structure in the off-state [37]. A planar periodic structure comprises identical unit cells; therefore, it is evaluated using Floquet ports and master–slave boundary conditions. The master–slave boundary conditions, also recognized as linked boundary conditions, were employed to simulate planar periodic structural surfaces. The proposed structure works in the off-state with the graphene Fermi energy level *E_F_* = 0. When *y*-polarized linearly polarized waves are incident, the co-polarized waves transmit with high efficiency in the operating frequency band of 0.35–1.75 THz, with a transmission greater than 0.80, whereas the cross-polarized waves transmit with essentially little efficiency and a transmission smaller than 0.30. Thus, most transmitted waves are co-polarized; there is little change in the polarization state of the transmitted waves relative to the incident waves, and the polarization rotator will not work in the off-state of the graphene grating.

To better illustrate the transmission characteristics in the on-state of the proposed polarization rotator based on graphene, the Stokes parameters were introduced to describe the polarization state of the transmitted wave with *y*-polarized incidence [38], as follows:

(10) $$S=\left(\begin{array}{l} S_{0}\\ S_{1}\\ S_{2}\\ S_{3} \end{array}\right)=\left(\begin{array}{c} E_{y}^{2}+E_{x}^{2}\\ E_{y}^{2}-E_{x}^{2}\\ 2E_{y}E_{x}\cos(\varphi_{diff})\\ 2E_{y}E_{x}\sin(\varphi_{diff}) \end{array}\right)$$

where phase difference *φ_diff_* = *φ_y_ − φ_x_*. Parameters *φ_y_* and *E_y_* represent the phase and electric field intensity of co-polarized transmitted waves with *y*-polarized incidence, respectively. Parameters *φ_x_* and *E_x_* represent the phase and electric field intensity of a cross-polarized transmitted wave with *y*-polarized incidence, respectively. The polarization rotation angle *α* was adopted to demonstrate the function of the polarization rotator, and the ellipse angle *χ* was adopted to characterize the linear polarization characteristic [19,39].

(11) $$\chi =0.5\arcsin(S_{3}/S_{0})$$

(12) $$\alpha =0.5\arctan(S_{2}/S_{1})$$

In order to meet the requirement of the 45° polarization rotation angle with *y*-polarized incidence, the Stokes parameters need to adhere to specific numerical conditions. To correspond to the linear polarization features, it is important to keep a value of *χ* = 0. Therefore, parameter *S*_3_ should be zero. In order to obtain the polarization rotation angle of 45°, parameter *S*_1_ must be close to 0. Considering the above conditions, to simultaneously achieve *S*_3_ = 0, *S*_2_ ≠ zero, and *S*_1_ = 0, the *φ_diff_* should be 0° or 180°; meanwhile, *E_x_* and *E_y_* should be greater than zero. In addition, to optimize efficiency, it is imperative to ensure that *E_x_* and *E_y_* are as close to each other as possible while also being as large as possible.

The proposed metal grating and graphene grating structures can achieve these desired Stokes parameters. When the graphene grating is in the on-state, it has the same effect as metal grating. Thus, metal wire gratings and graphene gratings will form a Fabry–Perot-like cavity structure [40], which will transmit the linearly polarized wave perpendicular to the graphene grating with a polarization rotation angle of 45°. When a *y*-polarized electromagnetic wave is incident on the proposed structure from the −*z* direction (as illustrated in Figure 3), it initially propagates through the front metal grating and subsequently traverses the dielectric layer to reach the graphene grating. A fraction of the transmitted *y*-polarized wave can transmit through the back graphene grating, where it converts to a 45-degree polarization transmitted wave. However, the remaining *y*-polarized component is reflected by the back graphene grating, where it is converted to both *x*- and *y*-polarization components before being reflected back to the front metal grating. Meanwhile, the *x*-polarized component of the reflected wave is obstructed and reflected by the front grating, experiences further interaction with the back graphene grating, and undergoes the polarization conversion process: a portion is converted into 45-degree polarization and transmitted through the back graphene grating, while another portion is reflected as *x* and *y* polarization waves. In contrast, part of the remaining *y*-polarized component of the reflected wave can penetrate through the front grating, and part of the component will also be reflected to reach the back graphene grating. Consequently, terahertz waves are reflected back and forth in the Fabry–Perot-like cavity, and the incident wave and multiple reflected waves undergo constructive interference and polarization coupling, which increases the transmission amplitude of the desired polarization components.

**Figure 5:**
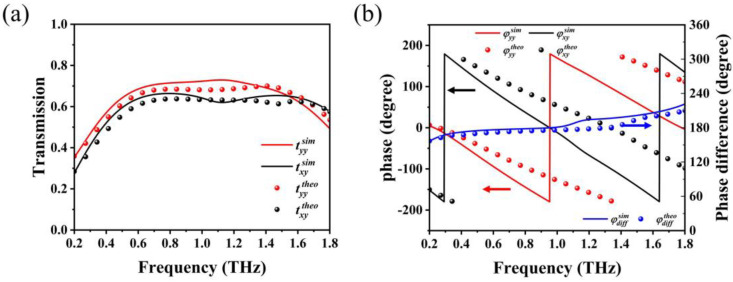
Simulated and theoretically calculated (**a**) transmittance of the co–polarization and cross-polarization with *y*–polarized incident waves in the on–state; and (**b**) phase of transmitted *x*– and *y*–polarized waves and the phase difference between them.

Figure 5a illustrates the simulated and theoretically calculated transmittance in the on-state for the graphene Fermi energy level of 1 eV, which are in reasonable agreement with each other. In the working frequency band, the transmission amplitudes of co-polarized and cross-polarized waves are nearly equal, and both are kept above 0.6. Figure 5b shows the phase *φ_yy_* of the transmitted co-polarized waves and the phase *φ_xy_* of the transmitted cross-polarized waves, as well as the phase difference between them. Both the simulated and theoretically calculated results show that the phase difference maintains nearly 180° in the whole working band, indicating that the generated transmitted waves are linearly polarized waves. There may be some inconsistencies due to errors in the MIT. Nevertheless, we believe that the differences observed fall within an acceptable range and can still be used to effectively verify the characteristics of the polarization converter.

**Figure 6:**
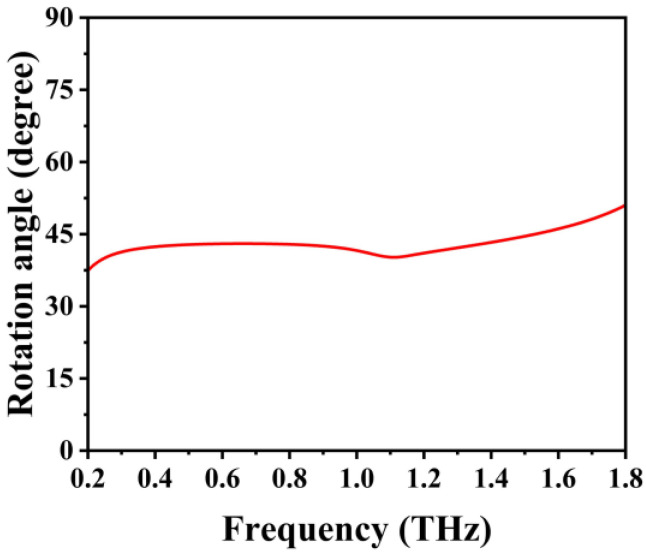
The polarization rotation angle of the transmitted wave relative to the polarization angle of the incident waves at Fermi level *E_F_* = 1 eV.

The simulated results meet the requirements of the desired Stokes parameters. The polarization rotation angle, which is calculated using Equation (10), is described as the angle by which the polarization direction rotates relative to the polarization direction of the incident waves. When the polarization rotator is in the on-state, the polarization rotation angle of transmitted waves with incident *y*-polarized waves of 0° is turned to nearly 45°. Within a frequency range of 0.35–1.75 THz, the polarization rotation angle of transmitted waves, as shown in Figure 6, was kept at around 45°. Hence, the proposed polarization rotator demonstrated the desired level of functionality.

To make it easier to analyze the performance of the polarization rotator, the *xoy* coordinate system in Figure 2 was rotated 45 degrees counterclockwise to achieve the *uov* coordinate system. The transmission amplitude in the *xoy* coordinate system can be defined as follows:

(13) $$t_{yy}=\left|E_{y}^{t}/E_{y}^{i}\right|$$

(14) $$t_{xy}=\left|E_{x}^{t}/E_{y}^{i}\right|$$

After the introduction of the *uov* coordinate system, the transmitted wave of *y*-polarized incidence can be decomposed into

(15) $$E_{y}^{t}=\vec{u}E_{u}^{t}+\vec{v}E_{v}^{t}$$

Thus, the transmission amplitude with *y*-polarized incidence in the *uov* coordinate system can be defined as:

(16) $$t_{uy}=\left|E_{u}^{t}/E_{y}^{i}\right|$$

(17) $$t_{vy}=\left|E_{v}^{t}/E_{y}^{i}\right|$$

Therefore, the polarization rotation performance of the device can be characterized by the following polarization conversion ratio (PCR) formula for *y*-polarized incidence, with *v* polarization being the desired polarization [41]:

(18) $$\mathrm{PCR}=\frac{{\left|t_{vy}\right|}^{2}}{{\left|t_{vy}\right|}^{2}+{\left|t_{uy}\right|}^{2}}$$

**Figure 7:**
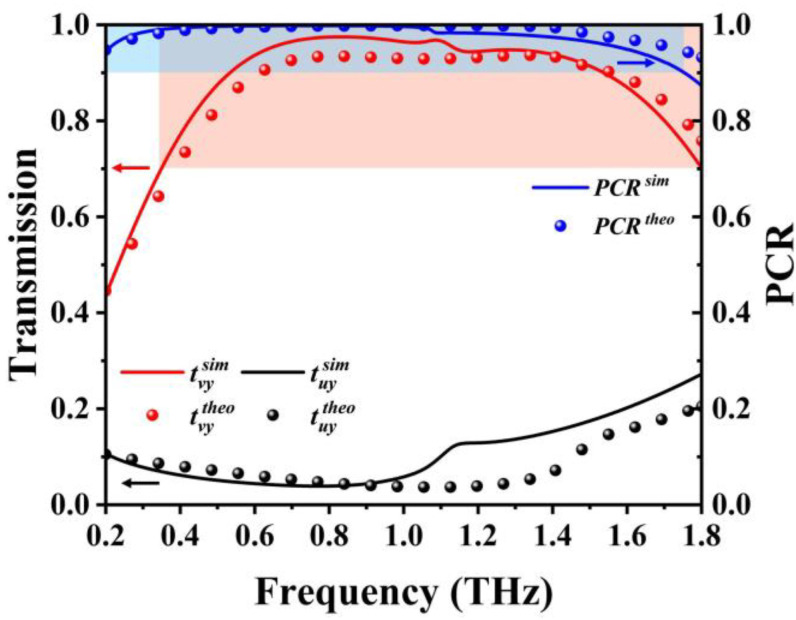
The transmission amplitude and PCR after coordinate conversion at Fermi level *E_F_* = 1 eV (sim: simulation results, theo: theoretical results).

Figure 7 shows the transmission amplitude and PCR that contain both simulation results and theoretical results after the transformation of the coordinate system. These results indicate that most *y*-polarized terahertz waves were rotated into polarized waves parallel to the *v*-axis, which is in reasonable agreement with theoretically calculated results. The simulated PCR remained above 0.9, within the frequency range of the blue region in Figure 7, and the 45-degree linear polarized transmission *t_vy_* remained above 0.7, within the frequency range of the red region. The operating frequency range of the proposed structure with good performance is the intersection of the red and blue regions, ranging from 0.35 to 1.75 THz, with the relative bandwidth reaching about 133.3%. Therefore, the proposed device can achieve a high linear polarization conversion efficiency over a wide operating bandwidth.

**Figure 8:**
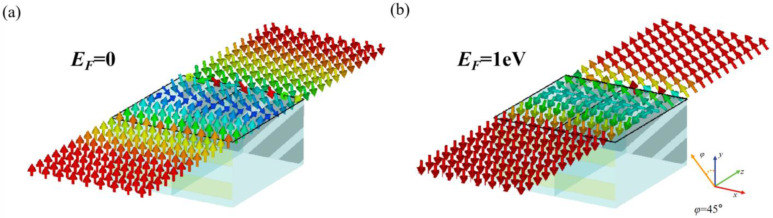
Electric field distributions: (**a**) graphene Fermi level *E_F_* = 0; (**b**) graphene Fermi level *E_F_* = 1 eV.

Figure 8 shows the schematic diagram of the electric field diagram at the central frequency. In the off-state of the reconfigurable polarization rotator, both the incident electromagnetic wave and the outgoing electromagnetic wave are *y*-polarized waves, as shown in Figure 8a. Figure 8b shows the electric field generated by the on-state polarization rotator. The transmitted wave has a rotation angle of 45° to the *y*-axis compared with the *y*-polarized incidence.

In conclusion, when a *y*-polarized terahertz wave is normal incident, the reconfigurable polarization rotator can switch the polarization angle between 0° and 45° by changing the Fermi level, and both operating states demonstrate good efficiency and a broad working band.

To show the advantages of the proposed polarization rotator, we compared it with some reported reconfigurable ones. The detailed comparison of the polarization converters proposed in the literature is presented in Table 1. Compared with other reported reconfigurable structures in [36,39,41,42], the most significant advantage of our structure is its ultra-wide operational bandwidth with high transmission amplitude and PCR. Moreover, the proposed polarization rotator is insensitive to oblique incidence, which increases its applicability and robustness.

### 3.2. Effect of Fermi Level on Polarization Converter

**Figure 9:**
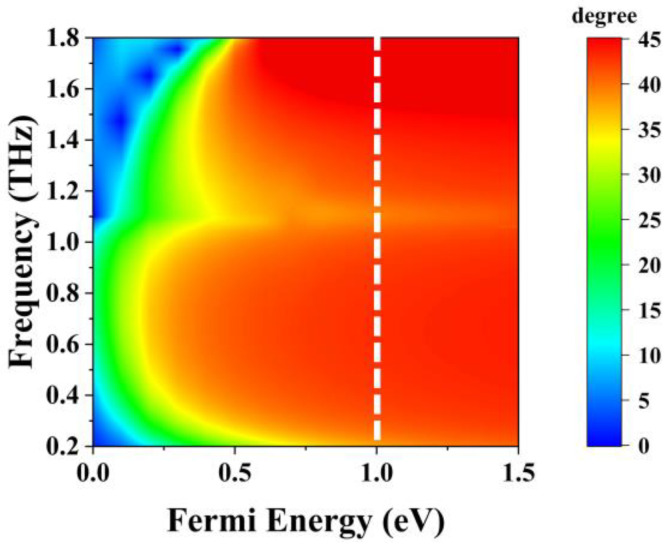
Polarization rotation angle at different Fermi levels.

The effect of the Fermi level on the performance of a reconfigurable polarization rotator has been investigated. The conductivity of the graphene grating at the Fermi level *E_F_* = 0 was close to zero when no bias voltage was applied to the graphene grating. Thus, the graphene grating is nearly transparent to terahertz waves. As shown in Figure 9, with the Fermi level increasing, the surface conductivity of the graphene grating increased and the polarization rotation angle gradually increased, indicating the increasing ability of polarization deflection. When the Fermi level *E_F_* ≥ 0.6 eV, the polarization rotation angle will be 45° over a wider bandwidth. However, an increase in the Fermi level resulted in a corresponding increase in the bias voltage. Too high a voltage cannot guarantee safety, and the Fermi energy levels usually range from −1 to 1 eV. For security and physical reliability, higher Fermi levels are unreasonable. The Fermi level *E_F_* = 1 eV, which is marked with a white-dotted line in Figure 9, has a wider operating bandwidth than the lower Fermi level; therefore, the Fermi level *E_F_* = 1 eV was chosen as the suitable working state for the graphene grating, which is the on-state.

### 3.3. Oblique Incidence Insensitivity Performance

**Figure 10:**
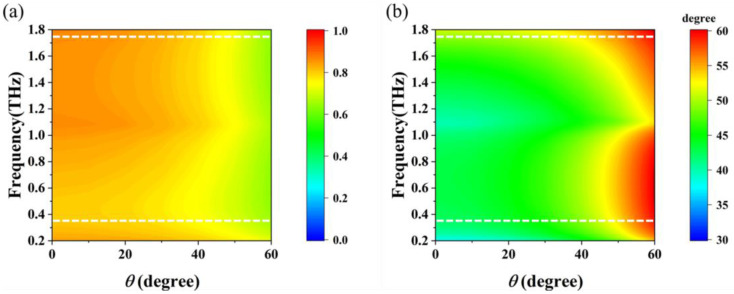
(**a**) Transmittance of the proposed structure at Fermi level *E_F_* = 0; (**b**) polarization rotation angle *α* of the proposed structure at Fermi level *E_F_* = 1 eV with incident *y*-polarized waves under different oblique angles *θ* (Inside the white-dotted line is the working band).

**Figure 11:**
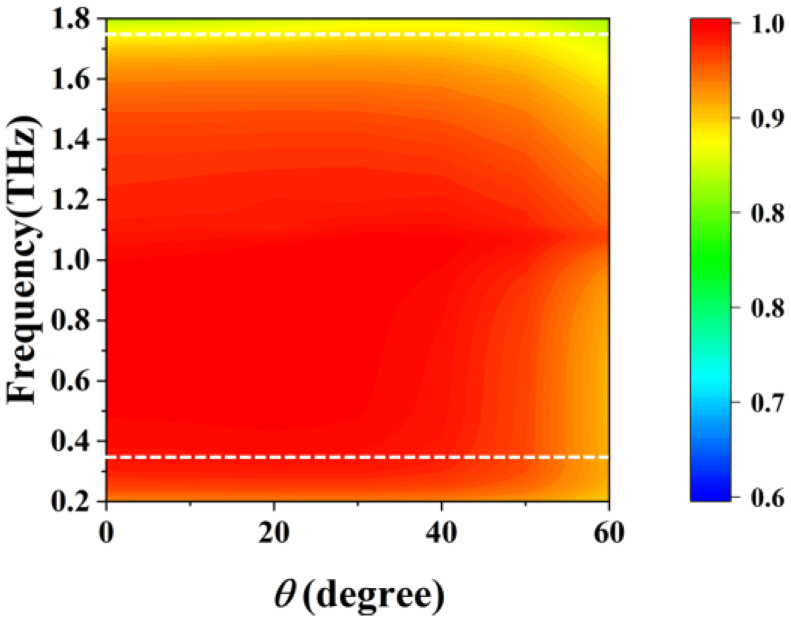
PCR at Fermi level *E_F_* = 1 eV with incident *y*-polarized waves under different oblique angles *θ* (Inside the white dotted line is the working band).

Terahertz waves encounter oblique incidence more frequently than perpendicular incidence; hence, the insensitivity of the reconfigurable polarization rotator to oblique incidence is also an essential indicator. Figure 10a shows the amplitude of transmitted *y*-polarized waves as the incident angle varied in the *xoz* plane, while the graphene grating was in the off-state with *y*-polarized incidence. When the incident angle was less than 50 degrees, the off-state structure exhibited a high transmittance above 0.7 for *y*-polarized waves with a frequency range of 0.3–1.75 THz, which is marked with the white-dotted line. When the graphene grating was loaded with a bias voltage, the polarization conversion angle *α* with different incident angles is shown in Figure 10b. In the on-state of the proposed polarization rotator with *E_F_* = 1 eV, as the incident angle *θ* varied, the polarization rotation angle remained at approximately 45° until the incident angle *θ* reached 50°, and the linear polarization characteristics remained stable in the working frequency band. When the incident angle *θ* was greater than 50°, the polarization rotation angle increased to 60°, which no longer met the design goal of the polarization rotator. The PCR in Figure 11 remained high when the incidence angle *θ* was less than 50° in the working band. Considering the periods of the metal and graphene gratings were 8 μm and 5.6 μm, respectively, which are about 0.028λ_0_ and 0.0196λ_0_, respectively, and much smaller (almost by two orders of magnitude) than the central working wavelength λ_0_ of incident terahertz radiation, the reconfigurable polarization rotator had better oblique incidence insensitivity over a relatively wide bandwidth.

## 4. Conclusions

The proposed reconfigurable polarization rotator based on graphene metamaterials has a simple structure and is a combination of metal gratings and graphene gratings, which can efficiently realize ultra-wideband polarization rotation of terahertz waves from 0.35 to 1.75 THz. Owing to the combination of anisotropic and cavity-like resonance-enhanced effects, the polarization rotation angle can be switched between 0° and 45° by changing the Fermi level of graphene.

When the bias voltage is applied to the graphene grating, the polarization rotation angle of the linearly polarized wave can be rotated from 0° to 45°. The relative ultra-wide bandwidth of a high 45-degree linear polarized transmission (above 0.7) and a PCR greater than 90% can reach 133.3% of the central working frequency. In addition, the polarization rotator has a stable and efficient polarization conversion in an ultra-wideband range, with incident angles within 0° to 50°.

In summary, the polarization rotator proposed can provide switching polarization rotation of linearly polarized waves in a wide operating frequency band, which has potential application in the fields of terahertz wireless communication and imaging.

## Figures and Tables

**Table 1:** Comparisons with other reported reconfigurable works.

Refs.	Working Mode	Conversion Mode	Frequency Range	Relative Bandwidth
[36]	Transmission	LP-to-LP	0.39–1.22 THz (|*t_xy_*| > 0.7) (PCR > 90%)	103%
[39]	Transmission	LP-to-LP	0.6–2.0 THz (|*t_xy_*| > 0.636)	107.7%
[41]	Reflection	LP-to-LP	2.92–6.26 THz (PCR > 90%)	73%
[42]	Reflection	LP-to-LP	2.15–4 THz (|*r_xy_*| > 0.5, |*r_yx_*| > 0.5) (PCR > 80%)	60.16%
This work	Transmission	LP-to-LP	0.35–1.75 THz (|*t_vy_*| > 0.7) (PCR > 90%)	133.3%

## Data Availability

Not applicable.
